# Supplementary material for: Epidemiology and aetiology of moderate to severe diarrhoea in hospitalised patients ≥5 years old living with HIV in South Africa, 2018–2021: A case-control analysis
Source: PLOS Glob Public Health. 2023 Sep 8;3(9):e0001718. doi: 10.1371/journal.pgph.0001718 (PMC10490993; doi:10.1371/journal.pgph.0001718)
Supplement: S1 Table — (DOCX) [file pgph.0001718.s002.docx]

S1 Table: Pathogens included in molecular testing

| **Pathogen** | **Testing platform** |
| --- | --- |
| **Viruses** | |
| Norovirus GI | FTD viral gastroenteritis, TaqMan Array Cards |
| Norovirus GII | FTD viral gastroenteritis, TaqMan Array Cards |
| Human astrovirus | FTD viral gastroenteritis, TaqMan Array Cards |
| Rotavirus | FTD viral gastroenteritis, TaqMan Array Cards |
| Human adenovirus | FTD viral gastroenteritis, TaqMan Array Cards |
| Sapovirus | FTD viral gastroenteritis, TaqMan Array Cards |
| Enterovirus | TaqMan Array Cards |
| CMV | Monoplex PCR |
| **Bacteria** | |
| *Campylobacer coli/jejuni/lari* | FTD bacterial gastroenteritis, TaqMan Array Cards |
| *Clostridioides difficile* | FTD bacterial gastroenteritis, TaqMan Array Cards |
| *Escherichia coli* verotoxin positives | FTD bacterial gastroenteritis |
| *Salmonella* spp. | FTD bacterial gastroenteritis, TaqMan Array Cards |
| *Shigella* spp. | FTD bacterial gastroenteritis, TaqMan Array Cards |
| Enteroinvasive *Escherichia coli* | FTD bacterial gastroenteritis, TaqMan Array |
| *Yersinia enterocolitica* | FTD bacterial gastroenteritis, TaqMan Array Cards |
| EAEC | TaqMan Array Cards |
| EPEC | TaqMan Array Cards |
| ETEC | TaqMan Array Cards |
| STEC/VTEC | FTD bacterial gastroenteritis; TaqMan Array Cards |
| *E. coli* O157 | TaqMan Array Cards |
| **Parasites** | |
| *Plesiomonas shigelloides* | TaqMan Array Cards |
| *Entamoeba histolytica* | FTD enteric parasites, TaqMan Array Cards, SeeGene Allplex |
| *Cryptosporidium* spp. | FTD enteric parasites, TaqMan Array Cards, SeeGene Allplex |
| *Giardia lamblia* | FTD enteric parasites, TaqMan Array Cards, SeeGene Allplex |
| *Vibrio cholerae* | TaqMan Array Cards |
| *Helicobacter pylori* | TaqMan Array Cards |
| *Schistosoma* | TaqMan Array Cards |
| *Enterocytozoon* spp. | TaqMan Array Cards, SeeGene Allplex |
| *Strongyloides stercoralis* | TaqMan Array Cards, SeeGene Allplex |
| *Cytoisospora belli* | TaqMan Array Cards |
| *Blastocystis hominis* | SeeGene Allplex |
| *Dientamoeba fragilis* | SeeGene Allplex |
| *Cyclospora cayentanensis* | SeeGene Allplex |
| *Hymenolepsis nana* | SeeGene Allplex |
| *Ascaris lumbricoides* | SeeGene Allplex |
| *Taenia* spp. | SeeGene Allplex |
| *Trichuris trichiura* | SeeGene Allplex |
| *Ancylostoma duodenale* | SeeGene Allplex |
| *Enterobius vermicularis* | SeeGene Allplex |
| *Necator americanus* | SeeGene Allplex |
