# Supplementary material for: Epidemiology and aetiology of moderate to severe diarrhoea in hospitalised patients ≥5 years old living with HIV in South Africa, 2018–2021: A case-control analysis
Source: PLOS Glob Public Health. 2023 Sep 8;3(9):e0001718. doi: 10.1371/journal.pgph.0001718 (PMC10490993; doi:10.1371/journal.pgph.0001718)
Supplement: S2 Table — (DOCX) [file pgph.0001718.s003.docx]

S2 Table: Clinical presentation for cases among PLHIV, stratified by CD4+ cell counts

|  | **CD4+ cell count ^a^** | | | ***p*-value** |
| --- | --- | --- | --- | --- |
|  | **<200 cells/µl (n=70)** | **200-500 cells/µl (n=21)** | **>500 cells/µl (n=8)** |  |
| **Duration of symptoms before admission – median (IQR)** | 6 (3-10) | 7 (3.5-12) | 5.5 (3-7) | 0.822 |
| **Chronic/persistent diarrhoea** | 6 (8.6%) | 2 (9.5%) | 1 (12.5%) | 0.855 |
| **Weight loss** | 64 (91.4%) | 17 (81.0%) | 5 (62.5%) | **0.032** |
| **Fatigue** | 58 (82.9%) | 16 (76.2%) | 5 (62.5%) | 0.293 |
| **Nausea** | 6 (75.0%) | 15 (71.4%) | 6 (75.0%) | >0.99 |
| **Vomiting** | 48 (68.6%) | 14 (66.7%) | 6 (75.0%) | >0.99 |
| **Fever (current or history)** | 46 (65.7%) | 14 (66.7%) | 5 (62.5%) | >0.99 |
| **Abdominal pain** | 41 (58.6%) | 14 (66.7%) | 4 (50.0%) | 0.667 |
| **Respiratory symptoms** | 33 (47.1%) | 12 (57.1%) | 1 (12.5%) | 0.097 |
| **Chills** | 32 (45.7%) | 7 (33.3%) | 3 (37.5%) | 0.634 |
| **Headache** | 32 (45.7%) | 7 (33.3%) | 4 (50.0%) | 0.635 |
| **Dermatological symptoms** | 13 (18.6%) | 3 (14.3%) | 0 (0.0%) | 0.494 |
| **Arthralgia** | 11 (15.7%) | 6 (28.6%) | 0 (0.0%) | 0.171 |
| **Myalgia** | 11 (15.7%) | 1 (4.8)% | 0 (0.0%) | 0.334 |
| **Neurological symptoms** | 5 (7.1%) | 0 (0.0%) | 1 (12.5%) | 0.347 |
| **Dysentery ^b^** | 2 (2.9%) | 0 (0.0%) | 0 (0.0%) | >0.99 |
| **Painful swollen glands** | 2 (2.9%) | 0 (0.0%) | 0 (0.0%) | >0.99 |

^a^ CD4+ count within 12 months of enrolment only known for 99 of the 164 PLHIV included in molecular testing; ^b^ Dysentery defined as self-reported blood in the stool.
