# Supplementary material for: Epidemiology and aetiology of moderate to severe diarrhoea in hospitalised patients ≥5 years old living with HIV in South Africa, 2018–2021: A case-control analysis
Source: PLOS Glob Public Health. 2023 Sep 8;3(9):e0001718. doi: 10.1371/journal.pgph.0001718 (PMC10490993; doi:10.1371/journal.pgph.0001718)
Supplement: S3 Table — (DOCX) [file pgph.0001718.s004.docx]

S3 Table: Pathogen detection in specimens of cases among PLHIV, stratified by CD4+ cell count

|  | **CD4+ count** ^a^ **- n (%)** | | | ***p*-value** |
| --- | --- | --- | --- | --- |
|  | **>500 cells/µl (n=8)** | **200-500 cells/µl (n=21)** | **<200 cells/µl (n=70)** |  |
| **Any pathogen** | 5 (62.5) | 12 (57.1) | 59 (84.3) | **0.022** |
| **Virus** | 2 (25.0) | 5 (23.8) | 19 (27.1) | >0.99 |
| **Adenovirus** | 0 (0.0) | 5 (23.8) | 8 (11.4) | 0.170 |
| **Adenovirus 40/41** | 0 (0.0) | 1 (4.8) | 0 (0.0) | 0.293 |
| **Norovirus** | 0 (0.0) | 1 (4.8) | 6 (8.6) | >0.99 |
| **Norovirus GI** | 0 (0.0) | 0 (0.0) | 0 (0.0) |  |
| **Norovirus GII** | 0 (0.0) | 1 (4.8) | 6 (8.6) | >0.99 |
| **Enterovirus** | 2 (25.0) | 0 (0.0) | 7 (10.0) | 0.068 |
| **CMV** | 0 (0.0) | 0 (0.0) | 2 (2.9) | >0.99 |
| **Astrovirus** | 0 (0.0) | 0 (0.0) | 2 (2.9) | >0.99 |
| **Rotavirus** | 0 (0.0) | 0 (0.0) | 1 (1.4) | >0.99 |
| **Sapovirus** | 0 (0.0) | 0 (0.0) | 1 (1.4) | >0.99 |
| **>1 virus detected** | 0 (0.0) | 1 (4.8) | 5 (7.1) | >0.99 |
| **Bacteria** | 4 (50.0) | 8 (38.1) | 35 (50.0) | 0.638 |
| ***Shigella* spp.** | 1 (12.5) | 1 (4.8) | 12 (17.1) | 0.389 |
| ***Salmonella* spp.** | 0 (0.0) | 0 (0.0) | 7 (10.0) | 0.322 |
| ***C. difficile*** | 0 (0.0) | 1 (4.8) | 9 (12.9) | 0.450 |
| ***Campylobacter*** | 0 (0.0) | 0 (0.0) | 4 (5.7) | 0.695 |
| ***STEC*** | 0 (0.0) | 0 (0.0) | 0 (0.0) |  |
| ***ETEC*** | 0 (0.0) | 0 (0.0) | 2 (2.9) | >0.99 |
| ***EPEC*** | 0 (0.0) | 0 (0.0) | 6 (8.6) | 0.464 |
| ***EAEC*** | 1 (12.5) | 4 (19.1) | 8 (11.4) | 0.622 |
| ***O157*** | 0 (0.0) | 0 (0.0) | 2 (2.9) | >0.99 |
| **Plesiomonas** | 0 (0.0) | 1 (4.8) | 0 (0.0) | **0.293** |
| **Helicobacter pylori** | 2 (25.0) | 2 (9.5) | 4 (5.7) | 0.109 |
| **>1 bacteria detected** | 0 (0.0) | 1 (4.8) | 14 (20.0) | 0.131 |
| **Parasite** ^b^ | 2 (25.0) | 8 (38.1) | 39 (55.7) | 0.145 |
| ***Cystoisospora*** | 1 (12.5) | 4 (19.1) | 12 (17.1) | >0.99 |
| ***Cryptosporidium* spp.** | 0 (0.0) | 1 (4.8) | 19 (27.1) | **0.028** |
| ***Blastocystis*** | 1 (12.5) | 2 (9.5) | 5 (7.1) | 0.563 |
| ***Giardia* spp.** | 0 (0.0) | 1 (4.8) | 4 (5.7) | >0.99 |
| ***Enterocytozoon* spp.** | 0 (0.0) | 1 (4.8) | 6 (8.6) | >0.99 |
| ***Schistosoma*** | 0 (0.0) | 0 (0.0) | 1 (1.4) | >0.99 |
| **>1 parasite detected** | 0 (0.0) | 1 (4.8) | 8 (11.4) | 0.611 |
| **Mixed infections**  **Virus-bacteria** | 1 (12.5) | 4 (19.1) | 9 (12.9) | 0.793 |
| **Virus-parasite** | 1 (12.5) | 3 (14.3) | 12 (17.1) | >0.99 |
| **Bacteria-parasite** | 2 (25.0) | 5 (23.8) | 21 (30.0) | 0.032 |

^a^ CD4+ count within 12 months of enrolment only known for 99 of the 164 cases among PLHIV; ^b^ The following parasites were screened for but not detected: *Vibrio cholerae*, *Yersinia enterocolitica*, *E. histolytica, Strongyloides* spp., *Cyclospora, Hymenolepsis, Ascaris, Taenia, Trichuris, Ancylostoma, Enterobius, Necator, Dientamoeba.*
