# Supplementary material for: Epidemiology and aetiology of moderate to severe diarrhoea in hospitalised patients ≥5 years old living with HIV in South Africa, 2018–2021: A case-control analysis
Source: PLOS Glob Public Health. 2023 Sep 8;3(9):e0001718. doi: 10.1371/journal.pgph.0001718 (PMC10490993; doi:10.1371/journal.pgph.0001718)
Supplement: S4 Table — (DOCX) [file pgph.0001718.s005.docx]

S4 Table: Clinical presentation of cases among PLHIV, stratified by treatment

|  | **Antiretroviral treatment** ^a^ **- n (%)** | | | **Cotrimoxazole prophylaxis** ^a^ **- n (%)** | | |
| --- | --- | --- | --- | --- | --- | --- |
|  | **Yes (n=130)** | **No (n=26)** | ***p*-value** | **Yes (n=63)** | **No (n=88)** | ***p*-value** |
| **Duration of symptoms before admission – median (IQR)** | 5 (3-8) | 4.5 (2-10) | 0.489 | 5 (3-9) | 4 (3-8) | 0.410 |
| **Chronic/persistent diarrhoea** | 10 (7.7) | 3 (11.5) | 0.456 | 6 (9.5) | 7 (8.0) | 0.774 |
| **Weight loss** | 108 (83.1) | 19 (73.1) | 0.231 | 57 (90.5) | 67 (76.1) | **0.023** |
| **Fatigue** | 98 (75.4) | 19 (73.1) | 0.804 | 56 (88.9) | 61 (69.3) | **0.005** |
| **Nausea** | 92 (70.8) | 19 (73.1) | 0.813 | 43 (68.3) | 65 (73.9) | 0.451 |
| **Fever (current or self-reported history in the past 10 days)** | 89 (68.5) | 20 (76.9) | 0.391 | 43 (68.3) | 61 (69.3) | 0.889 |
| **Vomiting** | 89 (68.5) | 22 (84.6) | 0.153 | 42 (66.7) | 67 (76.1) | 0.200 |
| **Abdominal pain** | 83 (63.9) | 18 (69.2) | 0.600 | 42 (66.7) | 59 (67.1) | 0.961 |
| **Respiratory symptoms** | 59 (45.4) | 13 (50.0) | 0.667 | 29 (46.0) | 41 (46.6) | 0.946 |
| **Headache** | 50 (38.5) | 12 (46.2) | 0.464 | 30 (47.6) | 33 (37.5) | 0.214 |
| **Chills** | 39 (30.0) | 6 (23.1) | 0.477 | 32 (50.8) | 14 (15.9) | **<0.001** |
| **Arthralgia** | 32 (24.6) | 2 (7.7) | 0.069 | 13 (20.6) | 21 (23.9) | 0.640 |
| **Dermatological symptoms** | 21 (16.2) | 2 (11.5) | 0.768 | 10 (15.9) | 14 (15.9) | 0.995 |
| **Myalgia** | 14 (10.8) | 1 (3.9) | 0.469 | 10 (15.9) | 6 (6.8) | 0.075 |
| **Dysentery** ^b^ | 6 (4.6) | 1 (3.9) | >0.99 | 3 (4.8) | 4 (4.6) | >0.99 |
| **Neurological symptoms** | 5 (3.9) | 1 (3.9) | >0.99 | 4 (6.4) | 2 (2.3) | 0.236 |
| **Painful swollen glands** | 3 (2.3) | 0 (0.0) | >0.99 | 2 (3.2) | 1 (1.1) | 0.571 |

^a^ ART status known for 156 and cotrimoxazole use known for 151 of 164 cases among PLHIV; ^b^ Dysentery defined as self-reported blood in the stool.
