# Supplementary material for: Epidemiology and aetiology of moderate to severe diarrhoea in hospitalised patients ≥5 years old living with HIV in South Africa, 2018–2021: A case-control analysis
Source: PLOS Glob Public Health. 2023 Sep 8;3(9):e0001718. doi: 10.1371/journal.pgph.0001718 (PMC10490993; doi:10.1371/journal.pgph.0001718)
Supplement: S5 Table — (DOCX) [file pgph.0001718.s006.docx]

S5 Table: Pathogens detected in cases among PLHIV, stratified by treatment

|  | **Antiretroviral treatment** ^a^ **- n (%)** | | | **Cotrimoxazole prophylaxis** ^a^ **- n (%)** | | |
| --- | --- | --- | --- | --- | --- | --- |
|  | **No (n=26)** | **Yes (n=130)** | ***p*-value** | **No (n=88)** | **Yes (n=63)** | ***p*-value** |
| **Any pathogen** | 23 (88.5) | 94 (72.3) | 0.082 | 66 (75.0) | 49 (77.8) | 0.693 |
| **Virus** | 6 (23.1) | 32 (24.6) | 0.868 | 20 (22.7) | 16 (25.4) | 0.704 |
| **Adenovirus** | 3 (11.5) | 14 (10.8) | >0.99 | 9 (10.2) | 8 (12.7) | 0.795 |
| **Adenovirus 40/41** | 0 (0.0) | 1 (0.8) | >0.99 | 1(1.1) | 0 (0.0) | >0.99 |
| **Norovirus** | 3 (11.5) | 10 (7.7) | 0.456 | 6 (6.8) | 7 (11.1) | 0.354 |
| **Norovirus GI** | 0 (0.0) | 1 (0.8) | >0.99 | 1 (1.1) | 0 (0.0) | >0.99 |
| **Norovirus GII** | 3 (11.5) | 9 (6.9) | 0.423 | 5 (5.7) | 7 (11.1) | 0.240 |
| **Enterovirus** | 1 (3.9) | 8 (6.2) | >0.99 | 4 (4.6) | 4 (6.4) | 0.720 |
| **CMV** | 0 (0.0) | 3 (2.3) | >0.99 | 2 (2.3) | 1 (1.6) | >0.99 |
| **Astrovirus** | 0 (0.0) | 3 (2.3) | >0.99 | 0 (0.0) | 2 (3.2) | 0.172 |
| **Rotavirus** | 0 (0.0) | 1 (0.8) | >0.99 | 0 (0.0) | 1 (1.6) | 0.417 |
| **Sapovirus** | 0 (0.0) | 0 (0.0) |  | 0 (0.0) | 0 (0.0) |  |
| **>1 virus detected** | 1 (3.9) | 5 (3.9) | >0.99 | 1 (1.1) | 5 (7.9) | 0.083 |
| **Bacteria** | 16 (61.5) | 62 (47.7) | 0.197 | 44 (50.0) | 32 (50.8) | 0.923 |
| ***Shigella* spp.** | 5 (19.2) | 24 (18.5) | >0.99 | 18 (20.5) | 9 (14.3) | 0.329 |
| ***Salmonella* spp.** | 2 (7.7) | 10 (7.7) | >0.99 | 6 (6.8) | 5 (7.9) | >0.99 |
| ***C. difficile*** | 2 (7.7) | 10 (7.7) | >0.99 | 6 (6.8) | 6 (9.5) | 0.544 |
| ***Campylobacter*** | 1 (3.9) | 10 (7.7) | 0.692 | 4 (4.6) | 7 (11.1) | 0.202 |
| ***STEC*** | 0 (0.0) | 0 (0.0) |  | 0 (0.0) | 0 (0.0) |  |
| ***ETEC*** | 1 (3.9) | 3 (2.3) | 0.522 | 3 (3.4) | 1 (1.6) | 0.641 |
| ***EPEC*** | 2 (7.7) | 8 (6.2) | 0.673 | 6 (6.8) | 5 (7.9) | >0.99 |
| ***EAEC*** | 2 (7.7) | 18 (13.9) | 0.531 | 10 (11.4) | 8 (12.7) | 0.803 |
| ***O157*** | 0 (0.0) | 3 (2.3) | >0.99 | 1 (1.1) | 2 (3.2) | 0.571 |
| **Plesiomonas** | 0 (0.0) | 1 (0.8) | >0.99 | 0 (0.0) | 1 (1.6) | 0.417 |
| **Helicobacter pylori** | 4 (15.4) | 8 (6.2) | 0.117 | 6 (6.8) | 6 (9.5) | 0.544 |
| **>1 bacteria detected** | 2 (7.7) | 24 (18.5) | 0.252 | 11 (12.5) | 13 (20.6) | 0.178 |
| **Parasite** ^b^ | 12 (46.2) | 59 (45.4) | >0.99 | 33 (37.5) | 37 (58.7) | **0.010** |
| ***Cystoisospora*** | 4 (15.4) | 24 (18.5) | >0.99 | 10 (11.4) | 17 (26.9) | **0.014** |
| ***Cryptosporidium* spp.** | 5 (19.2) | 21 (16.2) | 0.773 | 12 (13.6) | 14 (22.2) | 0.168 |
| ***Blastocystis*** | 4 (15.4) | 6 (4.6) | 0.063 | 8 (9.1) | 2 (3.2) | 0.195 |
| ***Giardia* spp.** | 1 (3.9) | 5 (3.9) | >0.99 | 4 (4.6) | 2 (3.2) | >0.99 |
| ***Enterocytozoon* spp.** | 0 (0.0) | 10 (7.7) | 0.215 | 4 (4.6) | 6 (9.5) | 0.321 |
| ***Schistosoma*** | 0 (0.0) | 4 (3.1) | >0.99 | 3 (3.4) | 1 (1.6) | 0.641 |
| **>1 parasite detected** | 2 (7.7) | 10 (7.7) | >0.99 | 7 (8.0) | 5 (7.9) | >0.99 |
| **Mixed infections**  **Virus-bacteria** | 3 (11.5) | 20 (15.4) | 0.768 | 11 (12.5) | 11 (17.5) | 0.394 |
| **Virus-parasite** | 2 (7.7) | 19 (14.6) | 0.531 | 8 (9.1) | 11 (17.5) | 0.126 |
| **Bacteria-parasite** | 8 (30.8) | 34 (26.2) | 0.633 | 19 (21.6) | 22 (34.9) | 0.069 |

^a^ ART status known for 156 and cotrimoxazole use known for 151 of 164 cases among PLHIV; ^b^ The following parasites were screened for but not detected: *E. histolytica, Strongyloides* spp., *Cyclospora, Hymenolepsis, Ascaris, Taenia, Trichuris, Ancylostoma, Enterobius, Necator, Dientamoeba.*
