# Supplementary material for: Epidemiology and aetiology of moderate to severe diarrhoea in hospitalised patients ≥5 years old living with HIV in South Africa, 2018–2021: A case-control analysis
Source: PLOS Glob Public Health. 2023 Sep 8;3(9):e0001718. doi: 10.1371/journal.pgph.0001718 (PMC10490993; doi:10.1371/journal.pgph.0001718)
Supplement: Attachment — Submitted filename: Response to reviewers_PLoS GH_HIV GI_April23.pdf [file pgph.0001718.s007.pdf]

24 April 2023

The Editor

PLoS Global Health

Re: Response to reviewers comments on manuscript 'Epidemiology and aetiology of moderate to severe diarrhoea in hospitalised HIV-infected patients ≥5 years old in South Africa, 2018-2021: a case-control analysis'

Dear Editor and Reviewers,

Thank you for the insightful comments and suggestions for improving our manuscript. Kindly see responses to comments below:

Editor's comments -

'We noticed that you used "data not shown" in the manuscript. We do not allow these references, as the PLOS data access policy requires that all data be either published with the manuscript or made available in a publicly accessible database. Please amend the supplementary material to include the referenced data or remove the references.'

Response: This has been removed. The data used to calculate the duration of illness will be made available with the dataset as per recommendation by the reviewers and editor.

'We notice that your supplementary tables are included in the manuscript file. Please remove them and upload them with the file type 'Supporting Information'. Please ensure that each Supporting Information file has a legend listed in the manuscript after the references list.'

Response: The supplementary tables have been removed from the manuscript file and uploaded as part of 'Supporting Information'. The figure titles for the supplementary tables are included in the manuscript after the reference list.

'In the online submission form, you indicated that "The datasets generated and/or analysed during the current study are not publicly available as they include study data which has not yet been published. Data are available from the corresponding author on reasonable request". All PLOS journals now require all data underlying the findings described in their manuscript to be freely available to other researchers, either 1. In a public repository, 2. Within the manuscript itself, or 3. Uploaded as supplementary information. This policy applies to all data except where public deposition would breach compliance with the protocol approved by your research ethics board. If

your data cannot be made publicly available for ethical or legal reasons (e.g., public availability would compromise patient privacy), please explain your reasons by return email and your exemption request will be escalated to the editor for approval. Your exemption request will be handled independently and will not hold up the peer review process, but will need to be resolved should your manuscript be accepted for publication. One of the Editorial team will then be in touch if there are any issues.'

Response: The dataset will be made available as supplementary information.

'Please include a statement on the determination of the sample size for the study in the methods section.'

Response: A sample size statement has been included in the methods.

Reviewer #1*: 'Thank you very much for letting me review this paper by Johnstone et al. The study provides important insights into the pathogenic causes of diarrhoeal admissions in HIV-infected patients in South Africa. The study design and methods are appropriate and well-described, and the sample size is reasonable given the scope of the study. However, there are a few minor limitations and areas for improvement that should be considered.'*

• *'The authors use 'HIV-infected' to refer to people living with HIV, please note that this is considered (and is) discriminatory language. Please replace this with the term "people living with HIV" (PLHIV). This is considered more appropriate and person-centered language than "HIV-infected." The latter term focuses solely on the medical condition and can perpetuate stigma and discrimination towards individuals living with HIV. Non-PLHIV can simply be referred to as HIV-Negative.'*

Response: This has been corrected throughout.

• *'Line 146 starts with saying patients of all ages were enrolled but later on text suggests this was only >5 years, or rather those included in this particular study, please clarify this by adding a sentence that the data obtained from the ANDEMIA study for this analysis focused on >5 years. This correction will also benefit from an explanation on why < 5 were excluded. Line 155, what about the 5 and 6 year olds?'*

Response: ANDEMIA included patients of all ages however for the purposes of this HIV analysis, only those 5 years and older were included. This was because there were very few PLHIV among the younger cases and controls. This has been clarified in the methods.

Consent was obtained from caregivers for all patients younger than 18 years, however those between 7-17 were also asked to sign an assent form if they were able to. Patients younger than 7 years old were not requested to sign an assent form (as per the ANDEMIA protocol).

• '*Considering the importance of viral load in HIV studies, this reviewer only landed on the explanation about why viral load was not available for analysis later in the discussion. Please include this information in the methods section soon after you mention CD4, considering the first few paragraphs of the discussion use viral suppression to discuss findings on CD4.'*

Response: An explanation of viral load not being available for the majority of patients has been included with the description of data extraction for CD4 cell counts.

• *'Statistically speaking, you can not use the word 'Effects' in lines 265-266 for a CC study as you are not trying to establish causality with this study. Table S2 presents chisq associations and they should be referred to as such. Causal language should be avoided.'*

Response: 'Effect' has been replaced with 'association'.

•*' If this reviewer understood this very well, there were no differences in clinical presentation (Lines 267-269) between the different categories of CD4 and between treated and untreated, is there an explanation for this? Considering the untreated and those with low CD4 are expected to present worse?'*

Response: This is likely a sample size issue. With the available sample size, we were unable to detect a difference in clinical presentation for different categories of CD4 and between treated and untreated. This has been stated in the limitations with the sentence: 'The sample size particularly limited the comparison of clinical presentation and pathogen detection between CD4+ cell categories and treatment groups amongst PLHIV.'

• *'Still on above, while there was no difference in clinical presentations, there were differences in pathogen prevalence (i.e. more pathogens in PLHIV with low CD4,Lines 274-276), how should the two be married together? Keep in mind a layman reader.'*

Response: The available sample size likely allowed detection of differences in pathogens by CD4+ categories where these differences were relatively large. The differences in clinical presentation are likely to be much smaller (those presenting with diarrhoea will have similar symptoms regardless of comorbid conditions, CD4+ cell class etc) and as such would require a much larger sample size (which was unfortunately not available for this analysis) to detect these differences.

• '*The discussion could also benefit from acknowledgement of the limitations of the case control approach'.*

Response: A short discussion of some limitations inherent to case control studies has been included as suggested.

• *‘Please make sure all the abbreviations have been defined STEC,ETEC,EPEC etc.’*
Response: The *E. coli* abbreviations have been defined in Table 3.

Reviewer #2: *‘The authors have addressed an important area on understanding the etiology of diarrhea in HIV-infected adults. Big studies on the etiology of diarrhea in SSA has been done for the under-five age group and not adults. Although the sample size limited their analysis, the results presented are important. I recommend the manuscript for publication with minor corrections on the basis that the methodology section has not been presented in sufficient detail for the study to be replicated. More information or citations should be included for the following:’*

- *‘Stool specimen/ rectal swab collection? Who collected and how was the pre-analytical sample treatment done to ensure the integrity of the specimen.’*
  Response: Specimens were collected by the patient, nursing staff or caregiver of minor patients. Specimens were transported on ice and kept in the fridge before processing. Stool is known to be an ideal transport and storage medium for microbes. The Fast-Track diagnostic kits and all in-house PCR tests included internal controls (which detect human DNA) to ensure specimen integrity and validity of the extraction and PCR process. This has been stated in the methods.
- *‘Explain or cite the three laboratory tests that were done.’*
  Response: The manufacturers of the laboratory kits have now been included.

Reviewer #3*: This is a carefully done analysis that addresses important questions about the aetiology of diarrhea in high-HIV prevalence settings. However, I am not sure that it currently complies with the journal's data availability policy (https://journals.plos.org/globalpublichealth/s/data-availability). For reference, the author's data availability statement is: "The datasets generated and/or analysed during the current study are not publicly available as they include study data which has not yet been published. Data are available from the corresponding author on reasonable request." This conflicts with the journal's policy, which states "it is not acceptable for an author to be the sole named individual responsible for ensuring data access." Furthermore, the potential for future publications is not considered a valid reason to not share data under the journal's policy (see the "Unacceptable Data Access Restrictions" subsection). The authors should review and respond to both the journal's data availability policy and my suggestions below before resubmitting.’*

- *‘The abstract seems to be split into different sections/paragraphs corresponding to the Background, Methods, Results, and Conclusion. If these sections are truly intended to stand apart in the abstract as separate paragraphs, they should be labeled with subheaders.’*
  Response: The subheadings have been included as suggested.
- *‘In the last paragraph of the introduction, it may be useful to state the seroprevalence of HIV infection in South African adults, to further support the statement "South Africa currently has the highest HIV burden in the world" and enable comparisons with the figures given for Botswana and Zambia.’*
  Response: The seroprevalence of HIV infections in South African adults has been included.
- *‘The methods section should clarify that controls and cases were not explicitly matched on the basis of age, location, HIV-status, or any other characteristics.’*
  Response: It has been stated that ‘unmatched’ controls were enrolled (line 156).
- *‘On line 33, I would suggest inserting the word "was" between "screening" and "performed".’*
  Response: This correction has been made.
- *‘On line 58, "With" may be a more suitable word choice than "Despite" (if WaSH improvements led to a decline in disease from water-borne pathogens, we would expect food-borne pathogens to comprise a higher share of overall diarrheal illness). Furthermore, since "WaSH" is an umbrella term that covers not just clean drinking water but also interventions that should prevent food-borne illness (hand washing, segregation of sewage from agriculture, etc), maybe the term "WaSH" should be replaced with "access to safe drinking water" or something similar, if that's what the authors mean.’*
  Response: This statement has been corrected as suggested.

Please let me know should you require any further clarifications.

Kind regards,

Siobhan Johnstone
