# Supplementary material for: Epidemiology and aetiology of moderate to severe diarrhoea in hospitalised patients ≥5 years old living with HIV in South Africa, 2018–2021: A case-control analysis
Source: PLOS Glob Public Health. 2023 Sep 8;3(9):e0001718. doi: 10.1371/journal.pgph.0001718 (PMC10490993; doi:10.1371/journal.pgph.0001718)
Supplement: Attachment — Submitted filename: Response to reviewers_PLoS GH_HIV GI_July23.pdf [file pgph.0001718.s008.pdf]

28 July 2023

The Editor

PLoS Global Health

<u>Re: Response to reviewers comments on manuscript 'Epidemiology and aetiology of moderate to severe diarrhoea in hospitalised patients ≥5 years old living with HIV in South Africa, 2018-2021: a case-control analysis'</u>

Dear Editor and Reviewers,

Thank you for the insightful comments and suggestions for improving our manuscript. Kindly see responses to comments below:

Reviewers' comments -

- *Reviewer #2: In the methods section, indicate if "tests were done according to manufacturer's instruction" for the test kits used. If there were any modifications on the manufacturer's instructions then they should be spelled out, otherwise "tests were done according to manufacturer's instruction" should be indicated. I am however very satisfied with the revised version.*

  Response: The sentence 'tests were done according to manufacturer's instructions' has been added (line 174-175).

- *Reviewer #3: The author's revisions have definitely improved the paper. I only have a few minor suggestions, and recommend publication if these are addressed.*

  On line 129 in the intro, "highest HIV burden" should be changed to "largest PLHIV population" (if that is what the authors mean) to avoid confusion, as the previous paragraph cites slightly higher prevalence estimates for Botswana and Zambia (23.9% and 22% vs 19.5%).
  Response: This has been amended as suggested (line 128).

  The new sample size statement in the methods section is appreciated, but it should be amended to 1) show how this calculation was performed or cite the method/approach used 2) clarify whether the requirement is 450 cases and controls combined, or 450 of each. or 450

total. If the former, the authors should clarify whether their power calculation assumes a 1:1 ratio of cases and controls (a 225:225 split will obviously have more power than a 449:1 split).

Response: The sample size calculation has been amended as suggested and now reads: 'In order to detect an 11% difference in pathogen detection between cases and controls (as per GEMS results) at 80% power, a sample size of 450 cases and controls combined was required (assuming a 1:1 ratio of cases and controls) as calculated using chi-squared test to compare two independent proportions.' (lines 166-167)

At a relevant point in the text (possibly the "data management and statistical analysis" section of the methods), the authors should include a short sentence indicating that the data is available in the supplementary materials.

Response: This sentence has been added to the 'Data management and statistical analysis' section as suggested (lines 194-195).

Please let me know should you require any further clarifications.

Kind regards,

Siobhan Johnstone
